# Case Report: Acute Splenic Artery Thrombosis in a COVID 19, Postpartum Patient

**DOI:** 10.3389/fmed.2021.698627

**Published:** 2021-11-05

**Authors:** Sebastian Daniel Trancǎ, Oana Antal, Anca Daniela Farcaş

**Affiliations:** ^1^Surgery Department, “Iuliu Haţieganu” University of Medicine and Pharmacy, Cluj-Napoca, Romania; ^2^Department of Anaesthesia and Intensive Care, Emergency Clinical County Hospital, Cluj-Napoca, Romania; ^3^Department of Anaesthesia and Intensive Care, Clinical Institute of Urology and Renal Transplantation, Cluj-Napoca, Romania; ^4^Internal Medicine Department, ”Iuliu Haţieganu” University of Medicine and Pharmacy, Cluj-Napoca, Romania; ^5^Cardiology Department, Emergency Clinical County Hospital, Cluj-Napoca, Romania

**Keywords:** splenic artery thrombosis, splenic infarction, post-partum, COVID 19 complications, case report

## Abstract

The incidence of thromboembolic disease is reported to be high in SARS-CoV_2_ disease. Pregnancy, an already physiologically hypercoagulable state, associated to COVID 19, generates even more concern regarding the potentially increased risk of thrombotic events. The exact incidence of such complications is yet unknown, but there is data suggesting that coagulopathy and thromboembolism are both increased in pregnancies affected by COVID-19. Since the outbreak of the COVID 19 pandemics, the most common described thrombotic events associated with SARS-COV2 infection have been venous thromboembolism and disseminated intravascular coagulation, while arterial thrombotic events are less commonly described. Splenic infarction is a rare disorder that can be secondary to a hypercoagulable state. There are only few cases of splenic infraction described, but none with splenic artery thrombosis, in a post-partum patient, on therapeutic anticoagulation regimen. We present the case of a 31-year-old Caucasian, 26 weeks pregnant woman, with no prior medical history, admitted to the hospital with a severe form of COVID 19 pneumonia and who, during the course of the disease, developed a massive splenic infarction with splenic artery thrombosis.

## Introduction

Incidence of thromboembolic disease is reported to be high in SARS-CoV2 disease (1, 2). A multitude of organ systems can be affected by this life threatening complication (3).

We present the case of a 31-year-old, with life threatening COVID 19 pneumonia, who developed splenic artery thrombosis with a massive splenic infarction while being therapeutically anticoagulated.

## Case Description

A 31-year-old Caucasian, 26 weeks pregnant woman, with a BMI of 29, with no prior medical history, was admitted to the hospital with progressive dyspnea, fatigue, and dry cough, which started 5 days prior to hospital admission. Real-Time PCR tested positive for SARS-CoV-2 and CT scan showed COVID 19 pneumonia in 90% of the lung. Five days after admission she was transferred to the ICU for severe dyspnea and polypnea, with extreme hypoxemia, where shortly afterwards, she was intubated and mechanically ventilated. Respiratory parameters on controlled mechanical ventilation and continuous neuromuscular blocking agents (NMBA) administration showed severe hypoxemia, with a PaO_2_/FiO_2_ ratio of 78, with low compliance. The respiratory course of the disease was complicated by two episodes of tension pneumothorax, adequately drained, and ventilator associated pneumonia (VAP) treated according to cultures. Following treatment, the pulmonary status improved gradually, reaching extubation criteria 16 days after the initial intubation. During admission time the cardiac function was within normal range; vasopressor support was necessary during the septic episodes, in order to maintain a MAP > 70 mm Hg. The renal function remained normal. After extubation, the patient showed normal cognitive functions, but had signs of severe neuropathy and myopathy.

**Figure 1:**
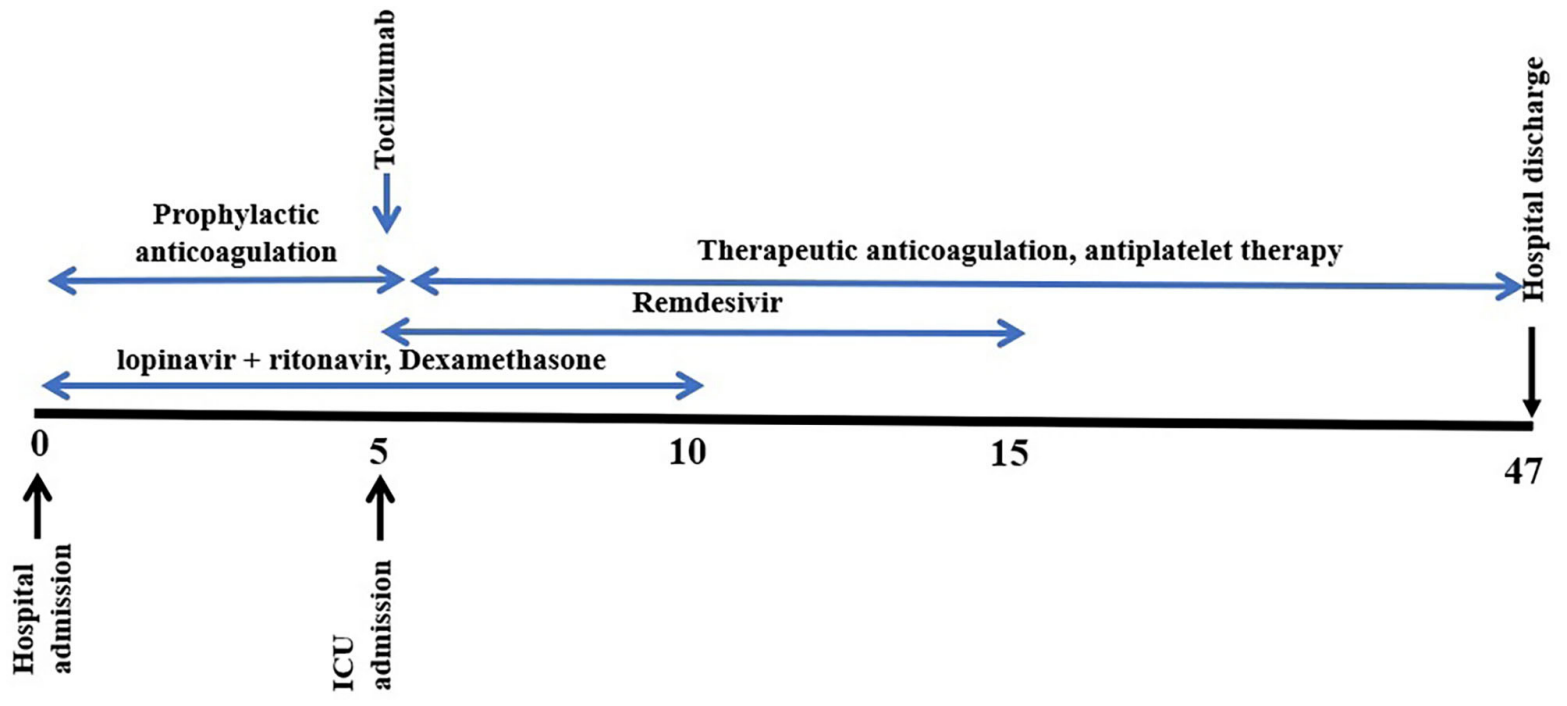
Treatment initiation time.

A 10-days course of Remdesivir was initiated at day 5 after admission, alongside Dexamethasone (started at the time of admission); therapeutic systemic anticoagulation with Enoxaparin (1 mg/kg, twice a day) and antiplatelet therapy (Aspirin) was also initiated at ICU admission time. Tocilizumab was also administered during early admission days, after an increased IL6 value was observed (5 days after admission). A 10-days course of lopinavir + ritonavir was initiated at the time of hospital admission (Figure 1).

The initially daily pregnancy follow up showed fetus viability, but with a gradually decreased umbilical cord blood flow. Ten days after her hospital admission, the intrauterine death of the fetus was revealed; a cesarean section was performed.

**Figure 2:**
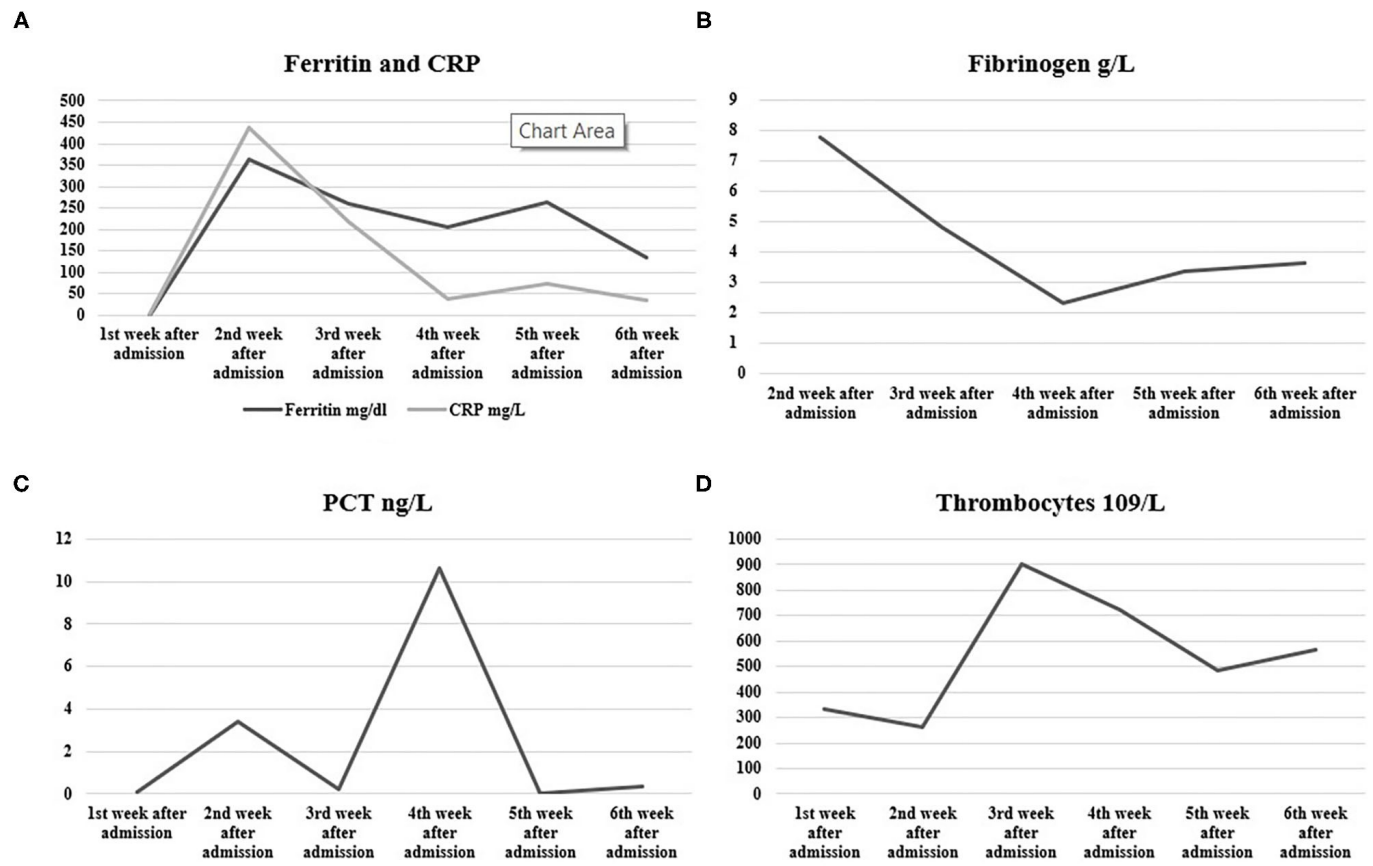
Dynamics of **(A)** C-reactive protein (CRP) and Ferritin, **(B)** fibrinogen, **(C)** procalcitonin (PCT), and **(D)** thrombocytes during the course of the disease.

In Table 1 and Figure 2, here are the dynamics of lab test during hospitalization.

**Table 1:** Relevant laboratory findings during hospital stay.

	**1st week after admission**	**2nd week after admission**	**3rd week after admission**	**4th week after admission**	**5th week after admission**	**6th week after admission**
Ferritin mg/dl (normal range: male 12–300 ng/ml, female: 10–150 ng/ml)		363	261	205	264	135
Il_6_ pg/ml (normal range: <7 pg/ml)		339.48				
CRP mg/L (normal range: 9–10 mg/L)	3	435.7	218.9	36.3	72.4	33.1
PCT ng/L (normal range: <0.05 ng/L)	0,1	3.4	0.2	10.61	0.06	0.35
Presepsin pg/ml (range >1,000 pg/ml suggestive for sepsis)			1,318	268		
Leucocytes 10^9^/L (normal range: 4–9 × 10^9^)	10,6	50.04	25.77	18.26	18.4	17.2
Thrombocytes 10^9^/L (normal range: 150–450 × 10^9^)	335	261	902	721	484	565
Fibrinogen g/L (normal range: 2–4 g/L)		7.77	4.8	2.34	3.35	3.63
aPTT sec (normal range: 24,5–36,5 s)		24.1	24	26.4	23.3	24.9
PT sec (normal range: 9–12, 4 s)		15.9	17.1	14.5	16.7	15.7

*aPTT, activated partial thromboplastin time; CRP, C-reactive protein; Il_6_, interleukin 6; PCT, procalcitonin; PT, prothrombin time*.

**Figure 3:**
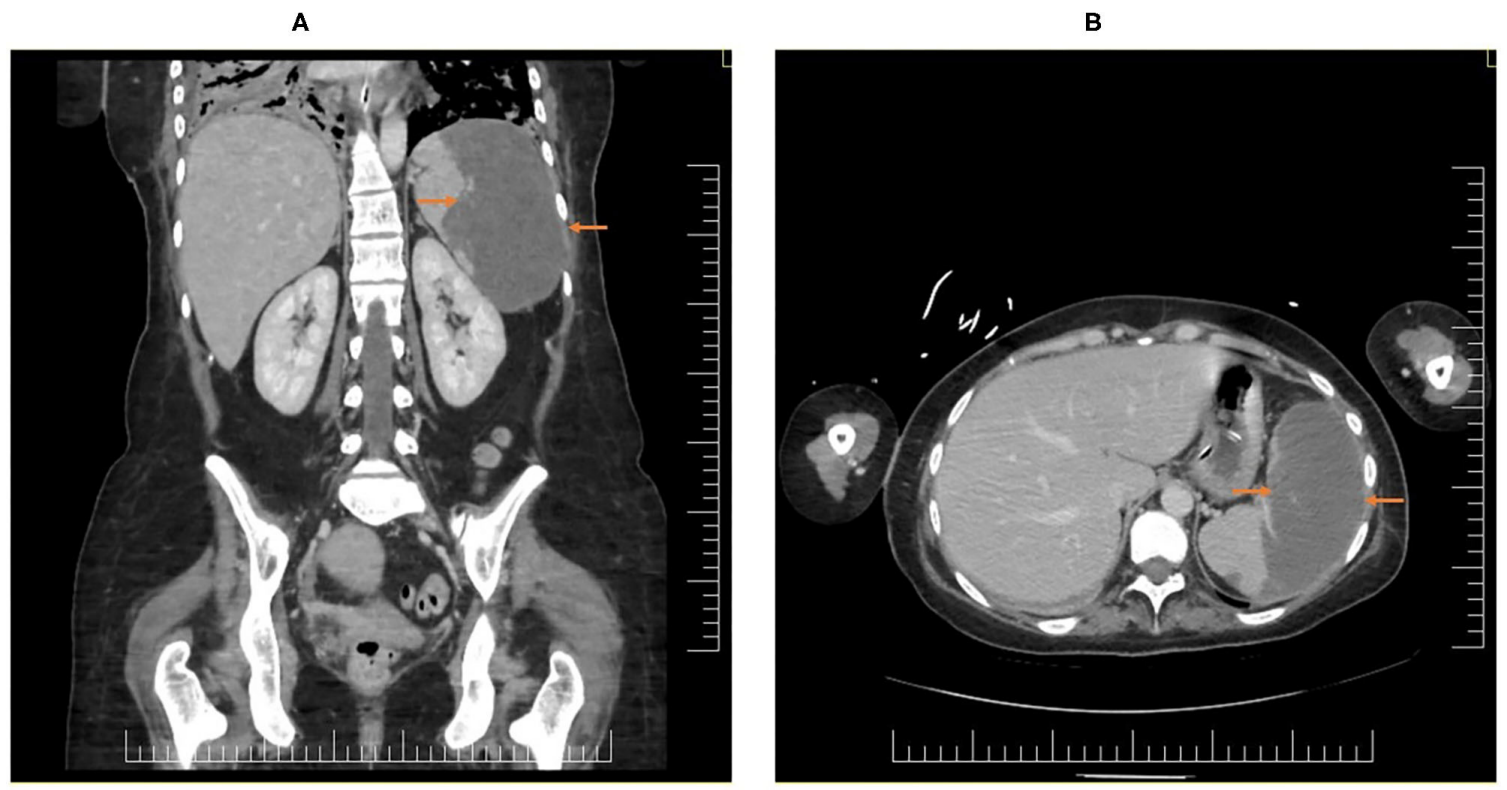
CT scan images showing the splenic infarction, **(A)** coronal plane and **(B)** axial plane.

During the course of the disease, she complained of mild, dull abdominal pain while developing progressive thrombocytosis; the clinical examination showed a soft, non-distended, non-tender abdomen. The CT-scan confirmed a 75% splenic infarction, with splenic artery thrombosis, with a major reduction in blood flow (Figure 3).

We would like to emphasize, that during her hospital stay, the patient was continuously on therapeutic anticoagulation (considered a high risk patient due to pregnancy and intrauterine death of the fetus) with mildly elevated D-dimer values, and fibrinogen levels within normal range, with the exception 1 week, when values ranged between 9 and 5 g/L. There were no thromboembolic or infarctions noted on a prior CT chest and abdomen done earlier in the admission period. Both the therapeutic anticoagulation with LMWH and the antiplatelet therapy were continued after the diagnosis of the thrombotic event.

## Discussion

Although initially described as a viral pneumonia, SARS-CoV-2 infection seems to present features of a multisystem disease, with impairment of several organs (4). Splenic infarction is a rare disorder that can be secondary to a hypercoagulable state (5). COVID-19 was associated with hypercoagulability and increased risk for venous and arterial thromboembolism (1–3). The underlying mechanisms of thrombo-embolic events during COVID 19 associated critical illness are not yet fully understood. The incidence of such complications was reported to be as high as 69%, in critically ill COVID 19, intubated and mechanically ventilated patients, under prophylactic or therapeutic anticoagulant dosage (6). Coagulation abnormalities may have a major impact on the outcome of COVID-19 patients (7).

Since the outbreak of the pandemics, the most common described thrombotic events associated with SARS-COV2 infection have been venous thromboembolism, but large-vessel ischemic stroke, acute upper or lower limb ischemia, abdominal and thoracic aortic thrombosis, mesenteric ischemia, myocardial infarction, and disseminated intravascular coagulation, have also been reported (8–14).

A recent systematic review on arterial thrombosis (AT) in COVID 19 patients, showed that most of the patients presenting AT were male, elderly, and had comorbidities (15). The anatomical distribution of arterial thrombotic events was shown to be wide, but none involved the splenic artery. The particularity of this case report relies on the fact that our patient was a young, healthy women, in post-partum period with an unusual site for AT.

While pregnancy is already a physiologically hypercoagulable state, there is concern regarding the potentially increased risk of thrombotic complications. The exact incidence is yet unknown, but there is data suggesting that coagulopathy and thromboembolism are both increased in pregnancies affected by COVID-19 (16). There are only few cases of splenic infarction described, but none with splenic artery thrombosis, in a post-partum, otherwise healthy patient, on therapeutic anticoagulation regimen. The described case highlight splenic infarction, with splenic artery thrombosis as a thrombotic complication of COVID-19.

Regarding the anticoagulation therapy after the diagnosis of the thrombotic event, we decided to continue the therapeutic dose of LMWH as prescribed by the hospital's cardiologist. This decision was made based on the recent COVID 19 guidelines on anticoagulation in VTE and PE (17), and also on the fact that there were no contraindication to LMWH.

## Conclusion

COVID-19 disease may be associated with AT in unusual sites, such as the splenic artery, even in patients on therapeutic anticoagulation.

## Data Availability Statement

The original contributions presented in the study are included in the article/supplementary material, further inquiries can be directed to the corresponding author/s.

## Ethics Statement

Ethical review and approval was not required for the study on human participants in accordance with the local legislation and institutional requirements. The patients/participants provided their written informed consent to participate in this study.

## Author Contributions

All authors listed have made a substantial, direct and intellectual contribution to the work, and approved it for publication.

## Conflict of Interest

The authors declare that the research was conducted in the absence of any commercial or financial relationships that could be construed as a potential conflict of interest.

## Publisher's Note

All claims expressed in this article are solely those of the authors and do not necessarily represent those of their affiliated organizations, or those of the publisher, the editors and the reviewers. Any product that may be evaluated in this article, or claim that may be made by its manufacturer, is not guaranteed or endorsed by the publisher.
